# Gut microbiota imbalances in Tunisian participants with type 1 and type 2 diabetes mellitus

**DOI:** 10.1042/BSR20182348

**Published:** 2019-06-18

**Authors:** Meriem Fassatoui, Mireia Lopez-Siles, Diana A. Díaz-Rizzolo, Haifa Jmel, Chokri Naouali, Ghaith Abdessalem, Asma Chikhaoui, Belén Nadal, Henda Jamoussi, Abdelmajid Abid, Ramon Gomis, Sonia Abdelhak, Margarita Martinez-Medina, Rym Kefi

**Affiliations:** 1 Laboratory of Biomedical Genomics and Oncogenetics, Institut Pasteur de Tunis, 13, Place Pasteur, B.P.74, 1002, Belvédère, Tunis, Tunisia; 2 University of Tunis El Manar, Campus Universitaire Farhat Hached, BP n° 94, Rommana, 1068, Tunis, Tunisia; 3 Laboratory of Molecular Microbiology, Biology Department, Universitat de Girona, Maria Aurèlia Capmany, 40, 17003, Girona, Spain; 4 Diabetes and Obesity Research Laboratory, Institut d’Investigations Biomèdiques, August Pi I Sunyer (IDIBAPS), Hospital Clinic, Rosselló 149-153, 08036, Barcelona Spain; 5 Department of Nutritional Diseases A, National Institute of Nutrition and Food Technology, 11, Rue Djebel Lakhdar, Bab Saâdoun, 1007, Tunis, Tunisia; 6 Department of External Consultation, National Institute of Nutrition and Food Technology, 11, Rue Djebel Lakhdar, Bab Saâdoun, 1007, Tunis, Tunisia

**Keywords:** Gut microbiota, qPCR, Tunisian, type 1 diabetes mellitus, type 2 diabetes mellitus

## Abstract

Gut microbiota plays an important role in the regulation of the immune system and host’s metabolism. We aimed to characterize the gut microbiota of Tunisian participants with and without diabetes.

We enrolled ten participants with type 1 diabetes mellitus (T1DM), ten patients with type 2 diabetes mellitus (T2DM), and 11 subjects without diabetes. Bacteria was quantified in fecal samples by quantitative PCR (qPCR). Statistical tests and multivariate analysis were performed using RStudio program.

Results showed that the proportions of Firmicutes, *Akkermansia muciniphila*, and *Faecalibacterium prausnitzii* (*P*≤0.041), as well as, the ratio Firmicutes/Bacteroidetes decreased in participants with T1DM compared with those without diabetes (p = 0.036). Participants with T2DM presented a reduction in the amounts of *A. muciniphila* and *F. prausnitzii* compared with those without diabetes (*P*≤0.036). Furthermore, *A muciniphila* is negatively correlated with glucose level (*P*=0.022) and glycated hemoglobin (HbA1c) (*P*=0.035). Multivariate analysis revealed that participants with diabetes formed a cluster apart compared with those without diabetes.

In conclusion the gut bacteria of Tunisian participants with diabetes was altered. The gut bacterial profile, especially the distribution of *A muciniphila* in participants with diabetes was affected by glycemic dysregulation. The investigation of the gut microbiota may help clinicians to improve diagnosis and treatment of diabetes and its complications.

## Introduction

According to the census of the International Diabetes Federation (IDF) in 2017, diabetes affects 425 million peoples aged from 20 to 79 years old worldwide [1]. In Tunisia, the prevalence of diabetes reaches 9.8 % [1].

Diabetes is characterized by an altered regulation of the metabolism associated to impaired insulin secretion and/or its action. Autoimmune destruction of β cells leads to type 1 diabetes requiring insulin injections [2]. In contrast, type 2 diabetes is defined by insulin resistance and usually preceded by overweight and obesity [1]. Diabetes could lead to several macrovascular and microvascular complications namely retinopathy, nephropathy, and neuropathy [2].

An altered composition of gut microbiota has been reported to be implicated in diabetes [3]. Individuals with type 1 diabetes mellitus (T1DM) presented a less stable and a little diverse intestinal microbiota, characterized by a rise in the amount of Bacteroidetes and a decrease in the abundance of *Bifidobacterium* and lactate and butyrate producing bacteria [4,5]. The low amount of butyrate producing bacteria such as *F. prausnitzii* identified in participants with T1DM, affects the physiological processes in the host [4]. The development of T1DM results from a dysbiotic intestinal microbiota leading to an alteration of the control of the gut mucosal barrier, facilitating the transit of antigens, and inducing aberrant immune reactions [6]. According to the literature early prescription of probiotics including *Bifidobacterium* and *Lactobacillus* could prevent autoimmune destruction of pancreatic β cells [7].

Changes in gut microbiota were revealed in type 2 diabetes mellitus (T2DM). Indeed, low abundance of butyrate producing bacteria and a decrease in the proportions of Verrucomicrobiae were shown [8]. In addition, a decrease in the amount of Firmicutes was described in participants with T2DM compared with those without diabetes [9]. An Iranian study indicated that the abundance of *Bifidobacterium* was reduced in participants with T2DM, compared with those without diabetes [10]. It was also reported that diet counseling re-established the profile of the altered gut microbiota of participants with T2DM by increasing the amounts of *F. prausnitzii* and *A. muciniphila* [10]. Noting that the abundance of *F.prausnitzii* was positively correlated with homeostasis and negatively associated with glycated hemoglobin (HbA1c) and fasting blood glucose (FBG) levels [11]. Furthermore, the abundance of *A. muciniphila*, which is a mucin degrading bacteria, was associated with the improvement of metabolic regulation [12].

The aim of the present study is to characterize the composition of gut microbiota of Tunisian participants, with and without diabetes, by evaluating the proportions of Firmicutes, Bacteroidetes, *A.muciniphila, F.prausnitzii*, and *Bifidobacterium spp*.

## Materials and methods

### Subjects characteristics, clinical diagnosis, and samples collection

We recruited 20 patients with diabetes (six female and four male with T1DM and six female and four male with T2DM) from the National Institute of Nutrition of Tunis. We gathered 11 participants without diabetes (eight healthy female and three male) from the Institut Pasteur de Tunis. Diabetes was diagnosed referring to the IDF criteria [1]. The participants were aged from 20 to 67 years old. For T1DM, autoantibodies to glutamic acid decarboxylase (GAD_65_) were also diagnosed [13]. Participants without diabetes had normal blood glucose levels. None of the enrolled subjects were under antibiotics treatment for at least 2 months before and at the time of stool sampling. Main clinical characteristics were summarized in Table 1. Anthropometric and clinical parameters were evaluated between participants with and without diabetes using *U* Mann–Whitney test.

**Table 1:** Sample size and clinical characteristics of subjects

Clinical characteristics	Participants without diabetes	Participants with T1DM	Participants with T2DM
Gender	Eight female	Six female	Six female
	Three male	Four male	Four male
BMIs (kg/m^2^) (mean ± SD)	23.32 ± 3.82	22.64 ± 3.72	28.77 ± 2.72
HbA1c (%) (mean ± SD)	4.8% (29 mmol/mol) ± 0.19	8.7% (72 mmol/mol) ± 1.83	8.4% (69 mmol/mol) ±1.83
Fasting plasma glucose (mean mmol/l ± SD)	5.08 ± 0.19	14.71 ± 5.98	11.10 ± 4

Abbreviations: BMI, body mass index; SD, standard deviation.

While participants with T1DM were under daily insulin therapy, participants with T2DM were under oral antidiabetic drugs treatment.

Fecal samples were collected from all subjects in sterile boxes and conserved at −80°C until being processed.

### DNA extraction and purification

Microbial DNA was extracted from 180 to 220 mg of fecal samples using the QiAamp Fast DNA Stool Mini Kit (Qiagen) following the manufacturer’s guidelines. DNA concentrations were measured with a NanoDrop ND-2000 Spectrophotometer (Thermo Scientific) and ratios 260/230 and 260/280 were assessed to check purity of the extracts. Extracted DNA were stored at −20°C until being analyzed.

### Selection of bacterial biomarkers for quantification

In the present study, gut bacteria namely Firmicutes, Bacteroidetes, *F. prausnitzii, A. muciniphila*, and *Bifidobacterium* spp. were quantified. Total bacteria was estimated for data normalization purposes.

### Standard curves for quantitative PCR

Genomic DNA of *F. prausnitzii* (DSM17677), *Bacteroides thetaiotaomicron* (VPI5482), *A. muciniphila* (ATCCBAA-835), and *Bifidobacterium adolescentis* (DSM 20083) were extracted from cultures using Wizard Genomic DNA Purification Kit (Promega, U.S.A.) following the manufacturer’s guidelines, and they were used to establish the quantification standard curves. Standard curves ranged from 10^2^ to 10^7^ 16S rDNA copies/reaction.

### Quantitative PCR assays

The quantitative PCR (qPCR) reactions were achieved by the means of Applied Biosystems Real-Time PCR System Device (7500, Applied Biosystems, Foster City, CA, U.S.A.). Briefly, absolute quantifications were carried out using TaqMan® Universal PCR Master Mix (Applied Biosystems, Foster City, CA, U.S.A.), SYBR®Green PCR Master Mix (Applied Biosystems, Foster City, CA, U.S.A.) and up to 50 ng of genomic DNA template as required for each assay. All primers and probes used in the present study, as well as, qPCR conditions were detailed in Supplementary Table S1. Automatic analysis settings were accomplished with the intention of determining the threshold cycle (C_T_) values. Data analysis were performed by means of 7500 system SDS software (version 1.4) supplied by Applied Biosystems Manufacturer.

### Data normalization and statistical analysis

Referring to quantitative analyses, the relative abundance of bacteria was calculated based on the proportions of the target gut microorganism and the amount of the total bacteria. The ratio Firmicutes/Bacteroidetes was also calculated.

Kruskal–Wallis method was adopted in order to compare the bacterial distribution between groups of subjects. Boxplots representing relative abundance of gut bacteria were generated by means of GraphPad PRISM version 7. Further pairwise comparisons were achieved through *U* Mann–Whitney test. We calculated correlations between the relative abundances of the studied gut bacteria and body mass indexes (BMIs), glucose level, and HbA1c using Spearman’s correlation test. Statistical analysis were performed using SPSS version 20. Significance levels were established for *P*<0.05. Multivariate analysis were accomplished by means of the RStudio tool.

## Results

### Clinical characteristics of patients

We found higher levels of HbA1c (*P*=0.001) and fasting plasma glucose (*P*<0.001) in both groups with diabetes compared with participants without diabetes. Participants with T2DM presented high BMIs compared with those without diabetes (*P*=0.002).

### Abundance of microbial biomarkers signature of diabetes dysbiosis

**Figure 1:**
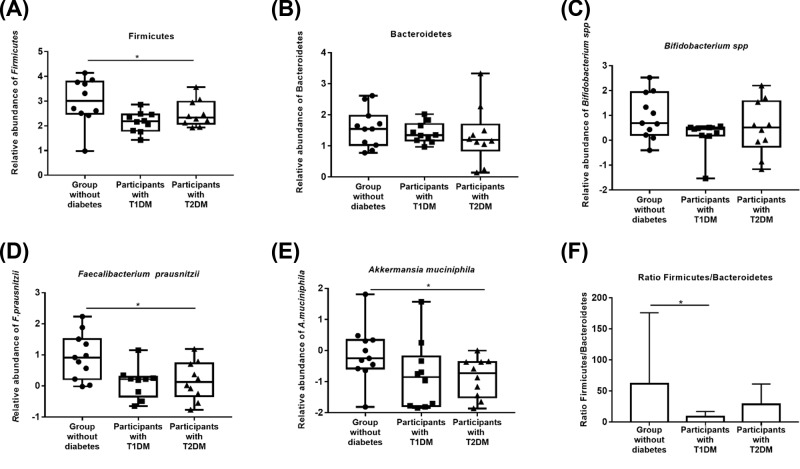
Box and whisker plots representing the relative abundance of bacterial biomarkers in participants with and without diabetes Medians were indicated with horizontal lines in the corresponding boxes. Data for the group without diabetes and for participants with T1DM and T2DM were shown. Relative abundance of Firmicutes (**A**), Bacteroidetes (**B**), *Bifidobacterium spp*. (**C**), *Faecalibacterium prausnitzii* (**D**), *Akkermansia muciniphila* (**E**), and the ratio Firmicutes/Bacteroidetes (**F**) in all groups were represented. Asterix were used to represent significant *P*-value (*P*<0.05).

The proportions of Firmicutes decreased globally in participants with diabetes compared with the group without diabetes (*P*=0.017) (Figure 1A). Differences were statistically supported between participants without diabetes and those with T1DM (*P*=0.01).

We did not observe any significant differences in the amounts of Bacteroidetes between participants with diabetes compared with those without diabetes (*P*>0.05) (Figure 1B). Assessment of the quantity of *Bifidobacterium spp* in participants with and without diabetes revealed and take off an approximately similar proportions of this bacteria between groups (*P*>0.05) (Figure 1C).

The loads of *F. prausnitzii* has been diminished in participants with diabetes compared with those without diabetes (*P*=0.034) (Figure 1D). Indeed, lower amount of *F. prausnitzii* were observed in participants with T1DM (*P*=0.03) and in those with T2DM compared with participants without diabetes (*P*=0.017).

Lower abundance of *A. muciniphila* was identified in the groups with diabetes (*P*=0.046) (Figure 1E). In this context, we detected a decrease in the load of *A. muciniphila* in participants with T1DM (*P*=0.041) and those with T2DM (*P*=0.036).

Finally, we calculated the ratio Firmicutes/Bacteroidetes, significant differences were detected between participants without diabetes and those with T1DM (*P*=0.036) (Figure 1F).

### Correlation between microbial biomarkers and clinical data

When considering all subjects together, no significant correlations were observed between the abundance of bacteria and BMIs (*P*>0.05) (Supplementary Table S2). The same results were revealed when we correlated HbA1c (Supplementary Table S2), serum glucose levels (Supplementary Table S2), and the amount of investigated gut bacteria except for *A. muciniphila*. Indeed, we reported a negative correlation respectively between glucose, HbA1c levels and the amount of *A. muciniphila* (*ρ*=-0.424, *P*=0.022 and *ρ*=-0.451, *P*=0.035)*.*

**Figure 2:**
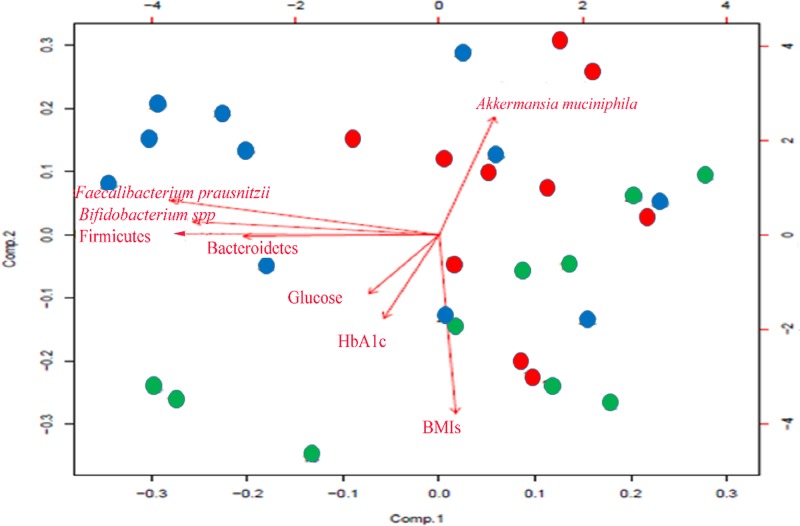
Multivariate analysis showing the distribution of variables across two components Participants with T1DM were represented by red circles. Participants with T2DM were represented by green circles. Participants without diabetes were represented by blue circles.

Multivariate analysis were performed (Figure 2) in order to assess clinical and/or bacterial variables influencing the distribution of gut bacteria. Participants with diabetes were clustered together whereas those without diabetes formed a distinguished cluster.

## Discussion

Gut microbiota was explored for the first time in Tunisian and North African participants with and without diabetes. We detected lower amounts of Firmicutes, *F. prausnitzii*, and *A. muciniphila* in participants with diabetes compared with those without diabetes. Nonetheless, no significant differences were observed in the proportions of *Bifidobacterium spp.* and Bacteroidetes between participants with diabetes and the group without diabetes. The unbalanced gut bacteria identified in Tunisian participants with diabetes was also reported on Chinese patients with T2DM, presenting a less diverse gut microbiota characterized by a reduction of butyrate producing bacteria [14]. Notably, a decrease in the proportions of *F. prausnitzii* in the intestine affects butyrate production, this product has an anti-inflammatory actions hampering; therefore, the activation of NF-κb signaling, modulating consequently the expression of inflammatory cytokines [15]; besides, butyrate consolidates gut integrity, and also regulates the survival and proliferation of enterocytes [16]. Moreover, butyrate production induces satiety and drop calorie intake through the control of glucagon-like peptide 1 (GLP1) and gastric inhibitory polypeptide production [16]. Furthermore, butyrate activates free fatty acid receptor 2, which is implicated in the regulation of insulin signaling pathway in adipose tissue and maintains energy homeostasis. Butyrate stimulates GLP 1 secretion in the gut, promoting the suppression of fat accumulation and improving sensitivity to insulin [15]. Indeed, alteration in intestinal microbiota profile could influence the regulation of glycemic metabolism leading to the pathogenesis of diabetes.

We observed that the ratio Firmicutes/Bacteroidetes is affected in Tunisian participants with T1DM compared with those with T2DM and without diabetes. It was reported that in mice, long chain inulin-type fructan fibers equilibrated the ratio Firmicutes/Bacteroidetes and protected against T1DM [17]. According to this experimental result, fibers intake equilibrated the gut microbiota and improved the control of glycemic metabolism, so it is recommended, especially for patients with diabetes, to consume more fibers in order to maintain a balanced intestinal microbiota for a better regulation of metabolism. As it was reported, the crosstalk between gut bacteria and the host immune system was mediated by microbiota-derived products controlling inflammatory responses. This interaction depends on the type of bacteria and on the metabolite produced and could be involved in β cell autoimmunity in T1DM [15].

We also revealed a decrease of the proportions of *A. muciniphila* in Tunisian participants with diabetes compared with the group without diabetes. This result was reported in prediabetics and newly diagnosed participants with T2DM [18]. It was described that a high abundance of this mucus-colonising specie was linked to a healthy state and improvement of glucose metabolization [19]. The administration of *A. muciniphila* to high fat diet mice for 4 weeks overcomed the metabolic endotoxemia [19], which is characterized by high plasma lipopolysaccharides-mediating inflammatory response [14] and counteracted insulin resistance [19]. This bacteria had beneficial effects by restoring mucus layer thickness and reducing weight gain in mice [19].

Unbalanced gut microbiota facilitates the development of certain bacteria and compromises the growth of others. In this context, it was reported that anaerobic bacterial fermentation of lactate by a certain type of bacteria leads to the production of acetate, succinate, and propionate. Consequently, the mucin synthesis is compromised by those products, tight junctions are altered, and intestinal permeability is affected [15]. Nevertheless, the same metabolite fermented by *Akkermansia* and *Prevotella* lead to the production of butyrate, which had beneficial effects on the host metabolism [15] .

In the present project, we revealed a significant negative association between the amount of *A. muciniphila*, glucose level, and HbA1c proportions. Nevertheless, no significant correlation was observed between the abundance of *F. prausnitzii, Bifidobacterium spp*., Firmicutes Bacteroidetes, and the aforementioned clinical parameters. Indeed, higher levels of HbA1c and glucose could be linked to a decrease in the proportions of *A. muciniphila* in Tunisian participants with diabetes. The failure of the control of glycemic regulation could be linked to a dysbiosis in gut bacteria. Estimation of the composition of the gut microbiota may help to elucidate the metabolic disorders and improve the control and prevention of diabetes and its complications.

In the present study, we determined an alteration of the composition of gut microbiota in Tunisian participants with diabetes compared with the group without diabetes. We detected a reduction in the amount of *A. muciniphila*, Firmicutes, and *F. prausnitzii* in Tunisian participants with diabetes, the abundance of *A. muciniphila* was also affected by glycemic level. Further investigations are required in order to better characterize gut microbiota in Tunisian participants with diabetes, taking into consideration other factors that might interact with gut bacteria, especially nutrition and lifestyle habits. This new area of research will help clinicians to think about new approaches to improve the management of diabetes.

## Abbreviations

ATCC: American Type Culture Collection
BMI: body mass index
DSM: Deutsche Sammlung von Mikroorganismen
GLP: glucagon-like peptide
HbA1c: glycated hemoglobin
IDF: International Diabetes Federation
NF-Kb: nuclear factor-kappa B
ρ: rho correlation coefficient of Spearman
T1DM: type 1 diabetes mellitus
T2DM: type 2 diabetes mellitus
VPI: Virginia Polytechnic Institute and State University, Blacksburg, VA, U.S.A
